# Myopia and Digit Ratio in Medical College Students

**DOI:** 10.1371/journal.pone.0089800

**Published:** 2014-02-21

**Authors:** Mathangi Krishnakumar, Shweta Atheeshwar, Mathangi D. Chandrasekar

**Affiliations:** 1 Department of Ophthalmology, Chettinad Hospital & Research Institute, Kelambakkam, Tamil Nadu, India; 2 Department of Physiology, Chettinad Hospital & Research Institute, Kelambakkam, Tamil Nadu, India; University of Goettingen, Germany

## Abstract

Myopia is amongst the most common refractive errors in the world. Both environmental and genetic factors are attributed to its causation, however all factors contributing to the development of myopia is yet to be found. Recent studies show presence of sex hormone receptor in the eyes. This has been shown to have a role in the development of various ocular pathologies. The second to fourth finger length ratio (2D:4D) has been hypothesised to be determined by exposure to sex steroids prenatally and thus considered a crude measure for prenatal androgen exposure. Hence this study was initiated to assess the association between myopia and 2D:4D ratio (a proxy marker to prenatal sex steroid exposure) among 100 medical college students of either sex and explore the possibility of role of prenatal sex steroids in causation of myopia. This study showed significant negative associations between myopia and digit ratio favouring a probable causal role of sex steroids on eye growth and development of myopia.

## Introduction

Myopia is the most common refractive errors in the world varying by country and ethnicity, reaching as high as 70–90% in certain Asian populations [1], [2]. In spite of numerous published works, search is on to identify various lesser known factors apart from environmental and genetic factors in the aetiology of myopia and excessive eye growth [3].

It usually occurs from a failure of the coordination of postnatal growth of whole eyeball with refractive components of the eye. The prevalence of myopia is higher in female as compared to males according to various population studies [4]–[6]. Myopia in particular is linked with enhanced mathematical ability, tendency of high IQ and left handedness [7]. The exact reason for this asynchrony and an explanation for myopia, laterality and IQ are lacking and it has been hypothesized that there may be a link between eyeball axial length and cerebral development, or that all may be influenced by the same genes and factors [8].

Prenatal testosterone has organizing effect on the brain and other organs. It causes masculization of the brain and increases in brain volume evident from twin studies conducted in male-female twins [9]. Lateralization is biologically programmed from the first day of life and it is immutable [10]. Several theories have been put forward to account for differences in lateralization, all in common perception that it occurs early in development in response to sex steroid exposure accounting for left handedness and high IQ [7], [11]. However the reason for association of myopia and handedness is not known. The idea of a common factor influencing brain and ocular growth in-utero is plausible. Recent research shows the presence of sex hormone receptor in the eye, thus a role of prenatal sex hormones on ocular growth and development can be hypothesised [12]. Studies also relate higher testosterone levels to increased prevalence and progression of myopia in females [13]. The second to fourth finger length ratio (2D:4D) has been hypothesised to be determined by prenatal sex steroids and thus considered a crude measure for prenatal androgen exposure [14]. It has been linked with various traits starting from left handedness, musical and mathematical abilities to increased sporting capabilities [14]. Manning et al hypothesized that all traits which are sexually dimorphic can be correlated with digit ratio [14].

The present study aimed to explore the relationship between myopia and 2D:4D ratio in Medical college students.

## Methodology

### 1. Subject Selection and Ethical Consideration

The study was initiated after obtaining clearance from the human ethical committee of Chettinad Hospital and Research Institute and written consent from the participants after explaining the study protocol. Undergraduate medical students in the age range of 19–21 years of both sexes were invited to participate in the study. A total of 235 medical students registered for the study. Students with history of previous surgeries for refractive error correction, neurological disease/disorder, long term medications and ocular pathologies like glaucoma and cataract were excluded.

### 2. Measurement of Refractive Error

The refractive errors of the participants were assessed by a trained optometrist using snellen’s chart and dioptres were calculated. Right eye was assessed followed by the left eye. The refractive error was measured without dilatation. In case of contact lens users, refraction was assessed after a 3 days lens free period. Mean of three values obtained by each method were taken as the final refractive power by that method. Spherical equivalent was calculated as sphere plus half negative cylinder. Myopia was defined as spherical error (SE) of at least −0.5 dioptres (D).

### 3. Measurement of Digit Ratio

A photograph of both hand were taken using a digital camera. The hands were held in supination with complete extension of fingers. All photographs were taken at same distances from midpoint of the hand. The lengths of the fingers were measured using tools in Adobe Photoshop [15]. Digit ratio, which is the ratio of the length of index and ring finger measured from the bottom crease to tip of the finger, was subsequently calculated. The mean ratio, which is the mean of the left and right digit ratio, was further calculated.

## Results

There were 200 subjects photographed and 125 subjects for whom refraction was measured. Both measures were available for only 100 subjects (male = 46, female = 54) and these data were used for analysis.

The data were analysed using SPSS version 17. Digit ratio in the study population was found to be between 0.99–1.00 (female : left hand-1.00±.050 Right hand- 0.99±.076, Mean- 0.99±0.051 and male: left hand- 0.981±0.09, right hand 0.993±0.05, mean 0**.**98±0.06) (Table 1). There was no sex difference in the digit ratio measured. Prevalence of myopia in the study population was found to be 54% (male = 54.3%, female = 53.7% (Table 2). The mean refractive error of right eye Male = 2.71±2.49D, Female = 2.37±2.22D and left eye Male = 2.54±2.14 Female = 2.56±2.14 showed no significant sex difference. Digit ratio (right, left and mean) of the myopes were lower when compared to the non-myopes, however a two-tailed t test showed a significant difference only in females (Table 3 and 4). Spearman’s correlation was used as to find association between digit ratio and refractive error. The refractive power of both the eyes showed a significant negative correlation with digit ratio as shown in Table 5.

**Table 1:** Digit ratio of male and female participants of the study.

	Right DigitRatio	Left DigitRatio	Mean DigitRatio
MALE (n = 46)	0.992±0.053	0.982±0.096	0.986±0.063
FEMALE (n = 54)	0.994±0.075	0.995±0.049	0.995±0.052
T Test	Not Significant	Not Significant	Not Significant

**Table 2:** Distribution of Myope among Male and Female participants of the study.

	MALE	FEMALE	Total
MYOPE	25 (54.3%)	29 (53.7%)	54%
NON MYOPE	21 (45.7%)	25 (46.3)	46%
TOTAL	46	54	100%

Values given are actual numbers and the values in parenthesis is percentage distribution.

**Table 3:** Digit ratio of Myope and Non myope in male subjects.

	MALE (n = 46)		
	MYOPE(n = 25)	NON MYOPE (n = 21)	T Test Significance
Right Digit Ratio	0.990±0.06	0.996±0.03	Not significant
Left Digit Ratio	0.968±0.07	0.997±0.12	Not significant
Mean Digit Ratio	0.979±0.07	0.996±0.06	Not significant

**Table 4:** Digit ratio of Myope and Non myope in Female subjects.

	FEMALE (n = 54)		
	MYOPE(n = 29)	NON MYOPE (n = 25)	T Test Significance
Right Digit Ratio	0.968±0.04	1.02±0.096	0.011
Left Digit Ratio	0.979±0.03	1.014±0.06	0.008
Mean Digit Ratio	0.976±0.03	1.018±0.06	0.002

**Table 5:** Spearman correlation between myopia And digit ratio.

	Spearman correlation Co-efficient	Significance
Right Digit ratio	−0.400	0.003
Left Digit ratio	−0.353	0.009
Mean Digit ratio	−0.408	0.002

## Discussion

The present study aimed to explore the relationship between myopia and 2D:4D ratio (a proxy marker of prenatal sex steroid exposure) among medical college students. The prevalence rate of myopia was found to be 54%. This prevalence is significantly higher when compared to the reported prevalence from India(6.9 to 19.7) [16]. This percentage is less in comparison to previous studies in Singapore (83%) and Taiwan (92.8%) medical students [1], [17]. However these findings are consistent with findings in Danish and Norway medical college students which showed 50% and 50.3% respectively [18]. There is no sex difference in the prevalence of myopia in the present study which is consistent with findings of other studies done in medical college students (13, 14). The different criteria for classification, methodology used to determine refractive error, non participation rates could attribute to variation in percentage among countries. Studies have shown correlation between higher intelligence, education levels and myopia [19]. Medical students being a select population of higher education levels could be a reason for high prevalence of myopia.

Animal experiments in study of myopia have shown that one of the possible modulator that trigger retinal, choroidal and sclereal growth is IGF-1(19). Recent studies show that *in utero* levels of IGF-I influence prenatal sexual development and IGF-I has a stimulating effect on gonadotrophin secretion, in turn increasing testicular testosterone secretion [20], [21]. Miller reported poor vision in opposite sex twins suggesting a hormonal hypothesis that increase in prenatal testosterone causes unfavourable environment *in utero,* stimulating proliferation of scleral tissue leading to myopia [22].

Manning et al., [23] hypothesised prenatal sex steroids affect the expression of HOX gene responsible for growth of digits and thus digit ratio (ratio of the lengths of index and ring finger) can be used as a window for *in utero* levels of sex steroids exposure [14] Higher prenatal testosterone exposure has been associated with lower digit ratio and vice versa [14]. Digit ratio is sexually dimorphic with male showing lower ratios compared to females. The present study showed no significant difference in digit ratio (right, left, mean) between males and females. The values were high compared to other studies in Indian population(male 0.96, female 0.97) [24]. Most of the studies have taken measure of right hand 2D:4D, hence data for comparison with left and mean are not available. Previous studies used manual measure with vernier calliper whereas the present study used digital photographs for measurement. This could account for the difference in values. The population size, geometrical distortion in photographs in comparison to manual measurement could have contributed to the result. Studies have shown that computer based measurement of digit ratio is superior to manual measurements [25]. A large population based study is required to determine the digit ratio of Indian ethnicity for comparison. The digit ratio (right, left, mean) was significantly lower in myopes when compared to non myopes among females. Faster progression and higher prevalence of myopia has been independently linked with the female sex (4). Studies have also shown higher testosterone levels in females with myopia this is corroborative to the result obtained in our current study which shows lower digit ratio in myopes among the female (13).

The absence of association between digit ratio and myopia in males can be explained by the occurrence of sexual dimorphism in polymorphism of steroidogenesis enzymes that influence sex steroid concentration in serum (13). Recent studies have shown the role of SMOC1 gene in the development of eye and limb by regulating the level of in utero sex steroid levels via the BMP (Bone Morphogenetic Protein) pathway (26). SMOC1 gene is particularly up regulated by testosterone (27). Male fetus show higher level of in utero concentration of testorene compared to female resulting in increased expression of SMOC1(27,28). Variations in SMOC1 expression have been associated with digit ratio (29). This sex difference in activity of gene in response to sex steroids and its effect on digit ratio may account for the difference in result between male and female.

The Geschwind-Behan-Galaburda (GBG) hypothesis states that prenatal sex steroids enhance right brain, cause changes in the patterns of brain lateralization and a shift to the left in handedness [26]. Left handedness is associated with increased abilities and is found more commonly among musicians, mathematicians, professional baseball and cricket players, architects and artists. Males show an increase tendency for left handedness and lower digit ratio [14]. Lower digit ratio has been linked with musical and mathematical abilities to increased sporting capabilities. Benbow found that left-handers and mixed-handers were over- represented among highly gifted adolescents who excelled in either mathematical or verbal reasoning ability. She also found increased prevalence of myopia among the gifted youths [27].

The present study consisted of medical college students supposedly representative of higher IQ group among general population and showed significant higher prevalence of myopia and negative correlation between digit ratio and myopia. i.e. lower digit ratio indicative of higher prenatal exposure to testosterone was associated with myopia. Thus role of in*-utero* levels of sex steroids in causation of myopia is plausible. This hypothesis is further justified by the fact that sex steroid hormone receptors are present in various ocular tissues, such as lens, retina, cornea, etc., [12]. Thus the association between myopia, left handedness and tendency of high IQ may be the result of common factor acting *in-utero* affecting cerebral development, eyeball growth and lateralization.

## Conclusion

In utero factors contributing to the development of myopia is plausible. Digit ratio can be used as a proxy marker to access the prenatal exposure to sex steroids. Studies relating digit ratio and various physical and physiological measures in Indian population have been initiated recently. A significant association between enhanced gross and fine motor skill [28] as well as swimming [29] among children in the age groups of 8–10 and 18–25 years respectively with lower digit ratio have been reported. Digit ratio being constant since birth [30] can be used to predict the risk for occurrence of myopia in a child at an early age warranting regular ophthalmological screening for children with lower digit ratio. Further research in this area in a wider population and age group will help ascertain the findings of the present study, on possibility of prenatal sex steroid exposure in pathogenesis of myopia.
